# Editorial: Community series in emerging frontiers in the formation of viable but non-culturable microorganisms and biofilms during food processing, volume II

**DOI:** 10.3389/fmicb.2023.1306559

**Published:** 2023-10-24

**Authors:** Zhenbo Xu, Yang Deng

**Affiliations:** ^1^Department of Laboratory Medicine, The Second Affiliated Hospital of Shantou University Medical College, Shantou, China; ^2^College of Food Science and Engineering, Qingdao Agricultural University, Qingdao, China

**Keywords:** food microbiology, viable but non-culturable state, biofilm formation, food safety and control, microbial cell viability

This Research Topic, entitled “*Community series in emerging frontiers in the formation of viable but non-culturable microorganisms and biofilms during food processing, volume II*,” was led by myself and Dr. Yang Deng. Between December 2021 to June 2023, this Research Topic has published six relevant articles. As the background of this topic was concerned, microorganisms, like all living organisms, naturally respond to changing environmental conditions. They display a remarkable ability to adapt to certain physical and chemical stresses in their environment. Survival mechanisms are activated following the detection of environmental signals and generate a complex adaptive response that leads to a state of tolerance and thus survival under sub-optimal or even sub-lethal conditions. When the environmental conditions threaten their survival or prevent them from living in optimal conditions, the cells are described as stressed. This notion of stress plays a fundamental role in the survival of microorganisms in foodstuffs.

The viable but non-culturable (VBNC) state, a unique state in which a number of bacteria respond to adverse circumstances, was first discovered in 1982 (Xu et al., 1982). The VBNC state, which has been extensively studied in bacteria, is characterized by an inability of the cells to grow on culture media, even though they are still viable and maintain a detectable metabolic activity (Liu et al., 2023). This state is reversible upon the return of favorable conditions. Various environmental factors can induce entry into the VBNC state including temperature, the physiological age of the culture, salinity, oxygen content, light, and ventilation. Most studies on VBNC cells have focused on pathogenic bacteria. Almost 100 bacterial species have been described as being able to enter into a VBNC state, both Gram-positive (e.g., *Listeria monocytogenes, Enterococcus, Micrococcus luteus*) and Gram-negative (e.g., *Escherichia coli, Vibrio cholerae, V. vulnificus, Legionella pneumophila, Campylobacter jejuni, Salmonella enterica, Pseudomonas aeruginosa, Helicobacter pylorii*) (Dong et al., 2020). In contrast, the VBNC state has received much less attention in other microorganisms, such as yeast and non-pathogenic foodborne bacteria.

In addition, just as all of us are challenged by the formation of biofilm on our teeth, a challenge that food processors and their sanitation teams face is the formation of biofilms on food equipment surfaces (Chitlapilly Dass and Wang, 2022). Food manufacturing plants, particularly sanitation and food safety/quality personnel, will want to recognize this potential hazard related to sanitation and that biofilms can have a profound impact on the safety and quality of their products. Biofilm formation can contaminate products through the introduction of pathogenic microorganisms or spoilage bacteria. They are difficult to remove, are often resistant to normal sanitation procedures, and can result in other detrimental process effects. Even when a food surface appears to be clean, the presence of biofilms is a potential hazard that must be eliminated and prevented from reoccurring. Before this can be done, it is important to understand what a biofilm is and how it is formed.

As a consequence, we proposed the current Research Topic, and aimed to provide an avenue for the dissemination of recent advances within the “VBNC” and “biofilms” fields, including (i) novel knowledge on induction and resuscitation mechanisms of VBNC microorganisms under food processing conditions and their biological characteristics, (ii) novel knowledge on mechanisms of biofilm formation and biofilm architecture under food processing conditions, and (iii) novel strategies and methods for the control of VBNC microorganisms and biofilms in food industrial settings.

Of the articles published in this Research Topic, two were about VBNC state, three were about biofilm, and one was a review, which were in accordance with our expectation and scope. The first published article in this Research Topic entitled “*Transcriptome Analysis of Viable but Non-Culturable Brettanomyces bruxellensis Induced by Hop Bitter Acids*” (He et al.), is about the VBNC state of a yeast which had received much less attention. The induction of a VBNC beer-spoilage yeast (*B. bruxellensis*) by hop bitter acids with different concentrations and its recovery is studied in this work. Moreover, transcriptome analysis is further performed to understand the mechanisms underlying the formation of hop bitter acids-induced VBNC *B. bruxellensis*. This study supplies a theoretical basis for microbial risk assessment in the brewing industry and focuses our attention on the VBNC state of yeast. Another article about VBNC state, entitled “*Formic acid, an organic acid food preservative, induces viable-but-non-culturable state, and triggers new Antimicrobial Resistance traits in Acinetobacter baumannii and Klebsiella pneumoniae*” (Yadav et al.), demonstrates for the first time that one of the widely used food preservatives, formic acid (FA), can induce a VBNC state at food processing, storage, and distribution temperatures (4, 25, and 37°C) with a varied time of treatment (days 4–10) in pathogenic Gram-negative bacteria *A. baumannii* and *K. pneumoniae*. Also, the removal of FA can resuscitate VBNC with an increased expression of multiple virulence and antimicrobial resistance genes in both pathogens. The authors propose that since food additives/preservatives are significantly used in most food manufacturing facilities supplying to hospitals, contamination of these packaged foods with pathogenic bacteria and the consequence of exposure to food additives emerge as pertinent issues for infection control and control of antimicrobial resistance in the hospital setting.

Concerning biofilm formation during food processing, two articles in this topic describe the control of biofilms and one studies its correlation with an antibiotic residual environment. The articles entitled “*Intense pulsed light for inactivating planktonic and biofilm molds in food*” (Li, Gu, Ye et al.) and “*Germicidal effect of intense pulsed light on Pseudomonas aeruginosa in food processing*” (Liang, Huang, Li et al.) both study the effect of intense pulsed light (IPL) on the biofilm formation of foodborne pathogens. The first study simulates the biofilm formed by *Aspergillus niger* and *Penicillium glaucum* in liquid and solid food in 96 well cell culture plates and polycarbonate membrane models, respectively, and investigates the fungicidal effect of IPL on planktonic and biofilm molds at three different capacitance parameters at room and refrigerator temperatures. This study describes that IPL could achieve over 90% fungicidal rates against planktonic and biofilm *A. niger* and *P. glaucum* at room and refrigerator temperatures. The other article studies the killing efficiency of different capacitances (650 μF, 470 μF, and 220 μF) of intense pulsed light on foodborne pathogenic microorganisms *P. aeruginosa* in the models of liquid food models, 96-well cell plates, and polycarbonate membrane models at room temperature (25°C) and refrigerated (4°C) environments to provide data to support the application of IPL sterilization devices in food processing. The bactericidal effect of IPL on *P. aeruginosa* is significantly influenced by the state of the bacterium. The larger the capacitance, the higher the number of pulses, and the better the sterilization effect on *P. aeruginosa*. Both studies prove that IPL is a promising non-thermal physical sterilization technique for fungal and bacterial inhibition on food surfaces. Another article about biofilm formation, entitled “*Biofilm formation of two genetically diverse Staphylococcus aureus isolates under beta-lactam antibiotics*” (Liang, Huang, Mao et al.), evaluates the biofilm formation of two genetically diverse *S. aureus* isolates under different concentrations of beta-lactam antibiotics on biomass content and biofilm viability. The two *S. aureus* isolates show resistance to beta-lactam antibiotics, penicillin, ampicillin, meropenem, streptomycin, and kanamycin. At certain sub-inhibitory concentrations, ampicillin (1/4 MIC), kanamycin (1/2 MIC), and streptomycin (1/4 MIC) can promote biomass accumulation, and 1/2 or 1/4 MIC penicillin and kanamycin can increase biofilm viability.

The last article in this Research Topic is a review entitled “*Pseudomonas aeruginosa: A typical biofilm forming pathogen and an emerging but underestimated pathogen in food processing*” (Li, Gu, Huang et al.). *P. aeruginosa* is a notorious gram-negative pathogenic microorganism because of its several virulence factors, biofilm-forming capability, and antimicrobial resistance. In addition, the appearance of antibiotic-resistant strains resulting from the misuse and overuse of antibiotics increases morbidity and mortality in immunocompromised patients. However, it has been underestimated as a foodborne pathogen in various food groups, for instance, water, milk, meat, fruits, and vegetables. This review covers recent advances in food safety related to *P. aeruginosa* including antimicrobial resistance, major virulence factors, and prevention measures. It is worth noting that food spoilage caused by *P. aeruginosa* should arouse wide concerns among consumers and food supervision departments.

It is noteworthy to point out that this series is a continuation of the previous Research Topic “*Emerging frontiers in the formation of viable but non-culturable microorganisms and biofilms during food processing*” which was also led by the same editorial team from June 2019 to December 2020, in which a total of 24 relevant studies and one editorial were published. In the previous editorial, entitled “*Editorial: Control of Viable but Non-Culturable (VBNC): important progress in VBNC study*,” we posed a significant controversy between VBNC cells and persisters. Recently, an important article touching upon this topic was published in Trends in Microbiology (Liu et al., 2023), and the authors clarified the definition of and differentiation between VBNC, persistent, dormant, and uncultured cells. However, despite clarification of such definition and differentiation, critical concerns still remain as to “how to define the microbial cell viability and how to accurately determine it.”

In conclusion, the above articles published in this Research Topic provide comprehensive knowledge of and insight into understanding the formation of VBNC state and biofilms during food processing, Which may aid in further control of such specific states of pathogens. Limitations of this Research Topic include a relatively small number of accepted articles (14 submitted and 6 accepted) and a lack of connected studies between VBNC state and biofilms despite this being one of the aims of this topic. Nevertheless, since the definition of VBNC has been clarified and differentiated from other similar terms such as persistent, dormant, and uncultured cells, research in the field of VBNC and biofilm under food processing has been advanced. In addition, we also look forward to important progress and articles to further touch upon the remaining critical concern between VBNC and other similar terms, which is “how to define the microbial cell viability and how to accurately determine it.”

## Author contributions

ZX: Writing—original draft, Writing—review & editing. YD: Writing—original draft, Writing—review & editing.
